# A Microcavity Array-Based 4D Cell Culture Platform

**DOI:** 10.3390/bioengineering6020050

**Published:** 2019-05-31

**Authors:** Cordula Nies, Tobias Rubner, Hanna Lorig, Vera Colditz, Helen Seelmann, Andreas Müller, Eric Gottwald

**Affiliations:** 1 Karlsruhe Institute of Technology, Institute of Functional Interfaces, Hermann-von-Helmholtz-P1atz 1, 76344 Eggenstein-Leopoldshafen, Germany; cordula.nies@kit.edu (C.N.); mail@hanna-lorig.de (H.L.); 2 Deutsches Krebsforschungszentrum, Im Neuenheimer Feld 280, 69120 Heidelberg, Germany; t.rubner@dkfz-heidelberg.de; 3 Bayer AG, Dept. Engineering and Technology, Kaiser-Wilhelm-Allee 3, 51373 Leverkusen, Germany; vera.colditz@outlook.com; 4 Roche Diagnostics GmbH, Nonnenwald 2, 82377 Penzberg, Germany; helense@gmx.de; 5 Städtisches Klinikum Karlsruhe, Frauenklinik, Moltkestr. 90, 76133 Karlsruhe, Germany; andreas.mueller.fk@klinikum-karlsruhe.de

**Keywords:** microcavity array, 3D cell culture, co-culture, hematopoietic progenitor cells, mesenchymal stromal cells, 4D microscopy platform

## Abstract

(1) Background: We describe a 4D cell culture platform with which we tried to detect and to characterize migration dynamics of single hematopoietic stem cells in polymer film microcavity arrays integrated into a microtiter plate. (2) Methods: The system was set up with CD34-expressing KG-1a cells as a surrogate for hematopoietic stem cells. We then evaluated the system as an artificial hematopoietic stem cell niche model comprised of a co-culture of human hematopoietic stem cells from cord blood (cord blood CD34^+^ cells, hHSCs) and human mesenchymal stromal cells (hMSCs) from bone marrow over a period of 21 days. We used a software-based cell detection method to count single hematopoietic stem cells (HSCs) in microcavities. (3) Results: It was possible to detect single HSCs and their migration behavior within single microcavities. The HSCs displayed a pronounced migration behavior with one population of CD34-expressing cells located at the bottom of the microcavities and one population located in the middle of the microcavities at day 14. However, at day 21 the two populations seemed to unite again so that no clear distinction between the two was possible anymore. (4) Conclusions: Single cell migration detection was possible but microscopy and flow cytometry delivered non-uniform data sets. Further optimization is currently being developed.

## 1. Introduction

Since the introduction of the niche concept by Schofield in 1978 [1], many niches have been identified although they are far from being completely understood. These stem cell niches are specialized microenvironments intended to keep the stem cell pool constant within a given organism throughout the entire life. This is achieved by the regulatory function of the niche that prevents the cells from entering the cell cycle and thereby protects the organism from stem cell exhaustion or from errors in DNA replication [1,2]. One of the most extensively studied niches is the hematopoietic niche, not only because of its potential in regenerative medicine applications but also because of the lack of information with regard to development, location(s), and regulation of the hematopoietic stem cell pool(s). Whereas primitive hematopoietic stem cells have been shown to reside in the yolk sac during early development of mice and humans [3], fully competent hematopoietic stem cells first arise from the aorta–gonad–mesonephros region as was shown by Medvinsky et al. [4] and Ivanovs et al. [5]. The development of hematopoietic stem cells (HSCs) continues with the migration into the fetal liver and subsequently into the bone marrow [3]. In the latter, it is assumed that at least two different niche compartments arise—the endosteal and the perivascular niche—of which the endosteal compartment harbors quiescent HSCs [6,7] and the perivascular harbors cycling HSCs [8]. A relatively new model, the “hemosphere model”, has been introduced by Wang and colleagues [9]. It can be characterized by clusters of HSCs in a zone between sinusoidal endothelial cells (SECs) and perivascular stromal cells (PVCs). In this model it was shown that the numbers of HSCs in bone marrow could be reduced when vascular endothelial growth factor receptor-2 (VEGFR2) from endothelial cells was deleted, thereby disrupting the formation of hemosphere structures. From this, it is obvious that the different niche compartments with their distinct functions have to be tightly controlled and regulated. Among the parameters influencing the niche microenvironment are the cellular composition, mechanical forces, soluble factors, oxygen tension, and more [10,11,12]. Many useful in vitro niche models have emerged during the last years shedding light on different aspects of the behavior of hematopoietic stem cells in their artificial environment, among which are static as well as dynamic models with various cellular compositions. Jing et al. [13] proposed a static two-dimensional model comprised of a co-culture of mesenchymal stromal cells and HSCs. Tan et al. [14] described a static biomimetic osteoblast three-dimensional niche model based on bio-derived bone and the effects on maintenance and expansion of hematopoietic stem/progenitor cells. Kurth et al. [15] described a static niche model based on adhesive microcavities with respect to marker expression and DNA-synthesis of hematopoietic stem and progenitor cells. Cook et al. [16] developed a static niche model that they termed micromarrows, which makes use of AggreWell^TM^ plates, and Wuchter et al. [17] proposed a dynamic niche model based on a microcavity array, consisting of a polymer film chip containing 634 microcavities (diameter of 300 µm) on an area of 10 × 10 mm and with outer measures of 20 × 20 mm. The chip was placed inside a microbioreactor that was actively super fused with medium, and was shown to largely maintain CD34-expression over time. Other microcavity models used much smaller diameters for single cell approaches that were based on polydimethylsiloxane (PDMS) [15] and analyzed the influence of spatial constraints or niche surface modifications on HSC fate [18,19,20]. 

We have developed a static artificial niche model, intended to shed some light into the behavioral dynamics of human HSCs, with respect to migration and proliferation, when the HSCs are in a three-dimensional co-culture together with human mesenchymal stromal cells. For this, the cells are cultivated in a microcavity array platform based on a 96-well microtiter plate footprint that comprises a variable number of microcavities and ensures a uniform size distribution of 3D aggregates cultivated therein [21]. The microcavity arrays are manufactured from 50 µm-thin polycarbonate films and are shaped via a microthermoforming technique that was developed earlier by Giselbrecht et al. and Truckenmüller et al. [22,23,24,25,26,27,28]. Typically, the dimensions of the formed microcavities are in the range of 300 µm in diameter and up to 300 µm in depth, depending on the application. 

For the establishment of the co-culture model, the first experimental series was intended to give insight into the proliferation and migration behavior of the CD34 expressing tumor cell line KG-1a to establish the methodology. After having developed the cell tracking and counting techniques, we introduced HSCs into the 3D environment, since the HSC population does not seem to be homogenous with respect to migration behavior, as was already shown by Jing et al. [13]. This group was able to discriminate HSCs in a co-culture system together with MSCs into three distinct subpopulations: non-adherent cells, cells adhering to the surface of mesenchymal stromal cells, and those beneath the mesenchymal stromal cells. Although the HSC population was homogeneous with regard to CD34 expression, they found differences in proliferation behavior and the associated cell cycle distribution. Moreover, slowly dividing cells underneath the MSC layer maintained CD34 expression much longer than the other two populations. We were therefore interested in the proliferation and dynamics of the migration of HSCs in the 3D context of microcavities and our goal was to establish a method to quantify the two parameters.

With the KG-1a cell line or hematopoietic stem cells from cord blood in co-culture with either the hepatocyte cell line Hep G2 or mesenchymal stromal cells from bone marrow, we established a confocal microscope-based method to detect proliferation and migration within a single microcavity volume, or even multiple microcavities at the same time depending on the magnification of the used objective. The KG-1a cell line has already been used by Francis et al. to establish a migration assay in 2D [29], but this has been done in a different context. We then tried to characterize the proliferation and migration behavior of human cord blood HSCs in co-culture with human mesenchymal stromal cells in this platform by applying open source, public domain algorithms for single cell tracking and counting. 

## 2. Materials and Methods 

### 2.1. Human Hematopoietic Stem Cell Isolation

Hematopoietic stem cells (hHSCs) were isolated from umbilical cord blood after obtaining informed consent according to the guidelines approved by the Ethics Committee of the Landesärztekammer Baden-Württemberg (F-2014-087). Mononuclear cells (MNCs) were isolated by density gradient centrifugation with the Ficoll-Hypaque technique (Biochrom KG, Berlin, Germany). CD34^+^ cells were purified by positive selection with a monoclonal anti-CD34 antibody using magnetic microbeads on an affinity column with the Magnetic Activated Cell Sorting system (AutoMACS system, all Miltenyi Biotec, Bergisch-Gladbach, Germany). The isolated cells showed a purity of >95% CD34^+^ cells.

### 2.2. Culture of KG-1a Cells

KG-1a cells (ATCC, CL-246.1, Manassas, VA, USA) were cultured under standard cell culture conditions. For routine passaging, 2 × 10^5^ cells in 5 mL culture medium (Iscove’s Modified Dulbecco’s Medium with 20% FCS) were transferred to a 25 mL culture flask. 

### 2.3. Co-Culture

**Figure 1:**
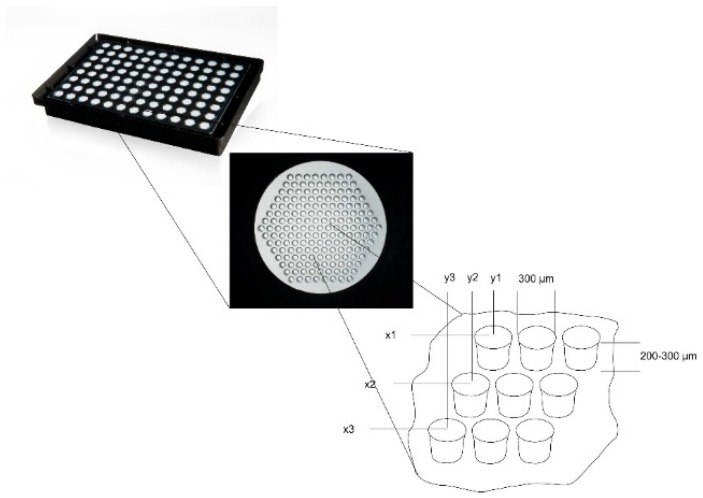
Microcavity array plate for the generation of 16,224 three-dimensional co-culture aggregates on a 96-well microtiter plate footprint. Each well of the microtiter plate contains an array of 169 microcavities; the cutout shows the principal layout of an array with x1y1, x2y2 illustrating the fixed position of each 3D aggregate as well as the diameter and depth of 300 µm and 200 to 300 µm, respectively.

For the establishment of the model, co-cultures of either 1.5 × 10^6^ Hep G2 (ATCC HB-8065, LGC Standards, Middleton, UK) in co-culture with 5 × 10^4^ KG-1a cells (ATCC CCL-246.1, LGC Standards, UK) or 7.5 × 10^4^ human mesenchymal stromal cells (hMSC) from bone marrow in co-culture with 5 × 10^4^ KG-1a cells or 5 × 10^4^ hHSC were used. Microcavity array plates (Figure 1, 300MICRONS GmbH, Karlsruhe, Germany) are made of polystyrene with a polycarbonate film forming the bottom of the plate that contains the microcavities. Prior to use, the plates were hydrophilized with a decreasing isopropanol series (100%, 70%, 50%, 30%, H_2_O) by pipetting 100 µL of the corresponding solution into each well for 30 s. After this, the wells were coated with a collagen I-solution (10 µg/cm^2^, approximated plate area: 28 cm^2^) and incubated at 4 °C over night. Prior to the inoculation, the cells were counted and pipetted into the wells with a standard pipette. The microcavity array plates were closed with a lid and incubated under standard cell culture conditions. Petri dishes (5 cm) with the same number of cells were cultured under the same conditions and served as 2D controls. 

### 2.4. Proliferation Assays

For the microscopic detection of proliferating cells, three different approaches were used: The labeling of DNA by thymidine-5’-ethynyl-2’-desoxyuridine (EdU), labeling the cytoplasm with CellTracker^TM^ Green (CTG), and labelling with carboxyfluorescein succinimidyl ester (CFSE). For confocal laser scanning microscopy the cells were fixed at the indicated timepoints of analysis. Each analyzed timepoint was performed as an individual experiment. Three microcavities were analyzed and at least three independent experiments of each indicated timepoint were carried out. Cell counts were determined according to the procedure described under Section 2.5. 

#### 2.4.1. Labeling of DNA

For the labeling of DNA in dividing cells the Click-iT-EdU chemistry (Click-iT^®^ EdU Imaging kit, Life Technologies, Darmstadt, Germany) was used. The assay is based on the introduction of a thymidine analog, the thymidine-5’-ethynyl-2’-desoxyuridine (EdU). By coupling of a fluorophore (AlexaFluor 488), DNA labeled in this way can easily be detected. The labeling of dividing cells was performed according to the manufacturer’s protocol. We used the Alexa Fluor^©^488 Imaging Kit (C10337, Life Technologies, Darmstadt, Germany). 

#### 2.4.2. Labeling with CellTracker^TM^ Green

The labeling of cells with CellTracker^TM^ Green (CellTracker™ Green CMFDA, Life Technologies, Darmstadt, Germany) is based on the uptake of 5-chloromethyl fluorescein diacetate (CTG) under physiological conditions. As soon as the cells have incorporated the molecule, the chloroethyl or bromomethyl groups react with thiol groups thereby rendering the molecule unable to pass through the membrane. By this, the cytoplasmic dye is imparted to the daughter cells. The labeling was performed according to the manufacturer’s protocol.

#### 2.4.3. Labeling with CFSE 

Cells can also be labeled with another cytoplasmic dye, the carboxyfluorescein succinimidyl ester (CFSE), which covalently binds intracellular amines. As with CellTracker^TM^ Green, the dye is localized cytoplasmically and is imparted to daughter cells upon cell division. We used CFSE of the CellTrace Cell Proliferation Kit (Life Technologies, Darmstadt, Germany) and stained the cells according to the manufacturer’s protocol.

### 2.5. Determination of the Position and Quantification of Cells in Z-Stacks 

For the quantification of proliferating cells in z-stacks the following steps were performed: After having stained and fixed the cells with the technique indicated in the corresponding section, z-stacks with an increment of 2 µm were made and imported into Fiji [30]. Only fully-depicted microcavities were used for cell counting, so partially-depicted microcavities were removed in all focal planes. To enhance contrast of the images and for better discrimination of the cells, the filter “unsharp mask” was used. Finally cells were counted with the plugin “3D Object Counter” [31]. The *Z*-coordinates of the counted objects were normalized to the depth of the microcavity. Because the depth of the microcavities can slightly vary from microcavity array (MCA) to microcavity array, whole depth was set to 100% and was divided into ten classes with equal step size (see Supplementary Figure S1).

The computed *Z*-coordinates of the counted objects were normalized to the depth of the microcavity according to Formula (1):

(1) $$Normalized\;distance\;to\;microcavity\;bottom\;=\frac{Z-coordinate\;of\;the\;counted\;object}{Depth\;of\;the\;microcavity}\cdot 100\%$$

To overcome potentially varying cell numbers from microcavity to microcavity, the amount of labelled cells per 10% of the image stack was normalized to the total number of labelled cells in the analyzed microcavity according to Formula (2):

(2) $$Normalized\;cell\;count=\frac{Number\;of\;labelled\;cells\;per\;10\%\;of\;the\;z-\;stack}{Total\;number\;of\;labelled\;cells\;per\;microcavity}\cdot 100\%$$

### 2.6. Lactate Dehydrogenase (LDH) Assay

The LDH assay (Cytotoxicity Detection Kit, Cat. no. 11644793001, Roche, Basel, Switzerland) was performed according to the manufacturer’s protocol. Briefly, 100 µL of culture supernatant was withdrawn from the culture, transferred to a microtiter plate and 100 µL of reaction mix was added. The absorption was read at 490 nm with a background subtraction at 650 nm. As a maximum control, a separate culture with the same number of cells was lysed with medium containing 2% Triton X-100. The results were expressed as % LDH of the maximum control. 

### 2.7. Microscopy 

Images were taken with a Leica TSC SP5 confocal laser scanning microscope (Leica Microsystems CMS GmbH, Am Friedensplatz 3, Mannheim, Germany). Microscope settings were kept fixed during each experiment. Distance between focal planes for every z-stack was 2 µm.

### 2.8. Immunofluoresence Stainings

At 1 to 21 days after seeding, the cells in the wells were carefully washed once with phosphate buffered saline (PBS) and subsequently fixed with 4% paraformaldehyde (PFA) for 15 min at room temperature. To stain for intracellular proteins, a permeabilization step with 0.2% Triton X-100 was performed. The following combinations of primary and secondary antibodies were used for indirect immunofluorescence staining: CD34 (CD34 mouse anti-human, (4H11(APG)), 10 µg/mL, ab762, Abcam, Bertha-Benz-Straße 5, Berlin, Germany) and goat-anti-mouse-Alexa Fluor 488 (AC-11001, ThermoFisher Scientific, 168 Third Avenue, Waltham, MA, USA) were used. In cases with propidium iodide counterstaining, a goat-anti-mouse-fluorescein isothiocyanate (FITC) conjugated antibody (62-6511, goat anti-mouse IgG (H+L) secondary antibody-FITC, ThermoFisher Scientific, 168 Third Avenue, Waltham, MA, USA) in combination with the primary antibody against CD34 (ab762) was used. For direct immunofluorescent staining and flow cytometry, a CD34 antibody labelled with allophycocyanine ((APC), CD34 mouse anti-human-APC (AC136-APC, ThermoFisher Scientific, 168 Third Avenue, Waltham, MA, USA) and a CD38 antibody labeled with FITC (CD38 Monoclonal Antibody (HIT2), 11-0389-42, FITC, eBioscience™, ThermoFisher Scientific, 168 Third Avenue, Waltham, MA, USA) were used. 

One well of a plate was incubated with 100–200 µL of the primary antibody solution from 1 h to overnight, depending on the readout, and then washed in PBS three times for 5 min each. Secondary antibody staining was performed by incubation in a 100–200 µL volume for 1 h at room temperature. After three additional washing steps, nuclear staining was performed with 500 µL Hoechst (1 µM, Hoechst 33342) or propidium iodide (1 µm, P3566, ThermoFisher Scientific, 168 Third Avenue, Waltham, MA, USA) for 5 min, followed by a final three washes in PBS for 5 min each. The samples were stored in PBS at 4 °C until microscope image acquisition.

Further processing and analysis of the images and composition of the image figures were performed with Fiji v1.52k. 

### 2.9. Flow Cytometry

**Figure 2:**
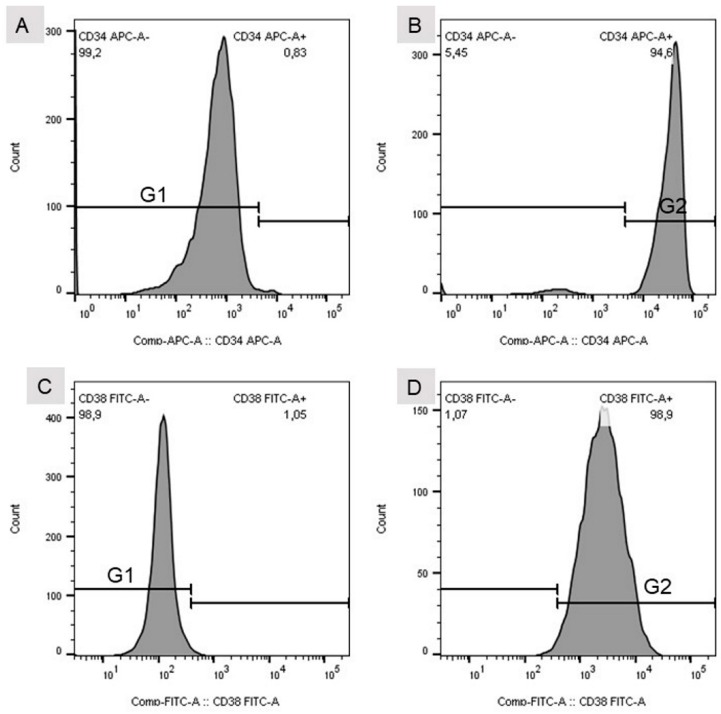

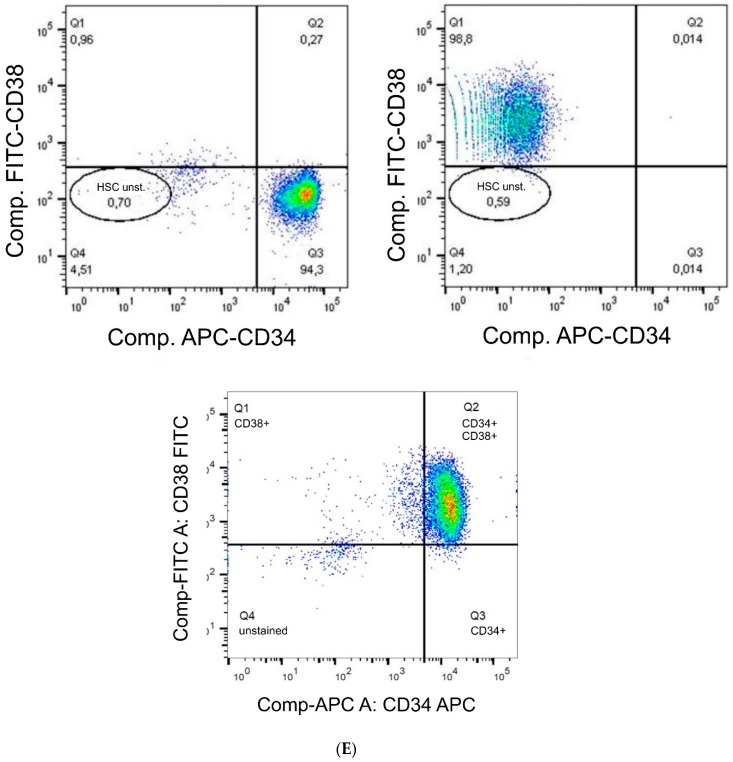
(**A**) Gate set for the isotype control of the CD34 mouse anti-human APC-labelled antibody. (**B**) Gate set for CD34 mouse anti-human APC-labelled antibody. (**C**) Gate set for the isotype control of the CD38 (HIT2)–FITC-labelled antibody. (**D**) Gate set for the CD38 (HIT2)–FITC-labelled antibody. (**E**) Detection of single (CD34^+^ or CD38^+^) and double positive (CD34^+^CD38^+^) stained cells. HSC unst. = unstained hHSC.

For flow cytometric analysis, a standard instrument was used (BD FACSVerse, BD Biosciences, 2350 Qume Drive, San Jose, CA, USA). Since FITC- and allophycocyanin-labelled (APC) antibodies were used for cellular analysis, excitation wavelengths were chosen to be 488 and 635 nm, and emission was analyzed at wavelengths of 530 and 660 nm, respectively. Fluorescence signals were analyzed with the help of BD FACSuite software (V.1.0.6, BD FACSVerse, BD Biosciences, 2350 Qume Drive, San Jose, CA, USA) and FlowJo software (V10, Tree Star LLC, Oregon, USA). Gates for cellular analysis were set according to the peak appearance of isotype controls and positive controls (Figure 2).

### 2.10. Statistical Analysis

The sample size for each experiment comprised at least three discrete samples for each co-culture experiment. Error bars in the diagrams indicate the standard deviation of the sample. The Mann–Whitney U-test was performed at the significance level 2α = 0.05.

## 3. Results

### 3.1. Establishment of the Model System: Migration and Proliferation Behavior of KG-1a Cells in Co-Culture with Hep G2 in Microcavity Arrays

**Figure 3:**
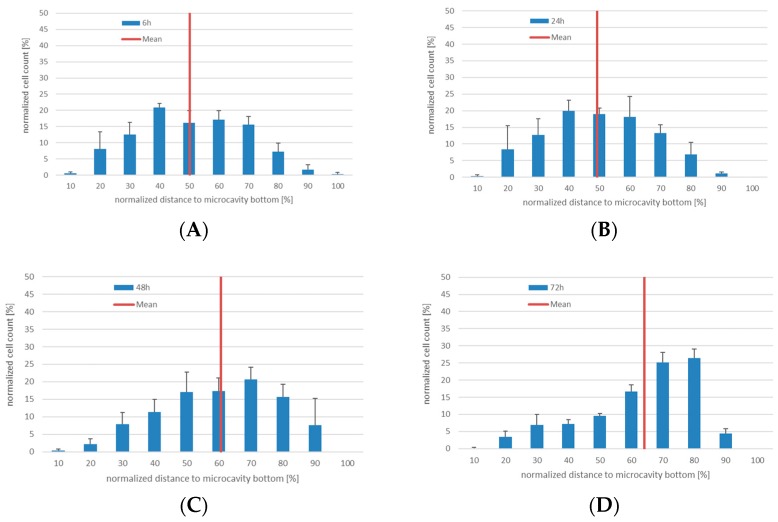
3D co-culture of KG-1a with Hep G2 cells in microcavities. Number of CellTracker^TM^ Green positive cells (CTG^+^) and their position relative to the cavity bottom (100%) at 6 (**A**), 24 (**B**), 48 (**C**), and 72 h (**D**), respectively. The mean is displayed as a red line, *n* = 3.

As a surrogate for CD34^+^ hHSCs, KG-1a cells were used to quantify the proliferation and migration behavior together with Hep G2 cells in 3D. For this, we used a 96-well-plate-based microcavity array system. Each well contained an array of 169 microcavities corresponding to 169 3D aggregates (see Figure 1). KG-1a cells in suspension culture were labeled with CellTracker^TM^ Green for two hours. After this, the cells were pooled with the Hep G2 cells and inoculated into the microcavity array. The cells were analyzed after six, 24, 48, and 72 h of culture (Figure 3, the microscope images are shown in Supplementary Figure S2. 

In 3D co-culture, the KG-1a cells displayed a pronounced migration behavior. Since the microcavities were completely filled with cells and no scaffold inside the microcavities was used, the observed migration of the cells was due to migration over the co-cultured cells that served as a “migration network”. Whereas for six and 24 h the median was 50% and 49%, after 48 and 72 h the median changed to 60% and 63%, respectively. Although not statistically significantly different, a tendency of a migration towards the bottom of the cavity can be seen. This behavior may indicate an intrinsic property of this niche model with regard to a minimum niche size that may be required for a niche to function properly [9]. 

### 3.2. Proliferation and Migration Behaviour of KG-1a in Co-Culture with hMSCs in Microcavity Arrays

**Figure 4:**
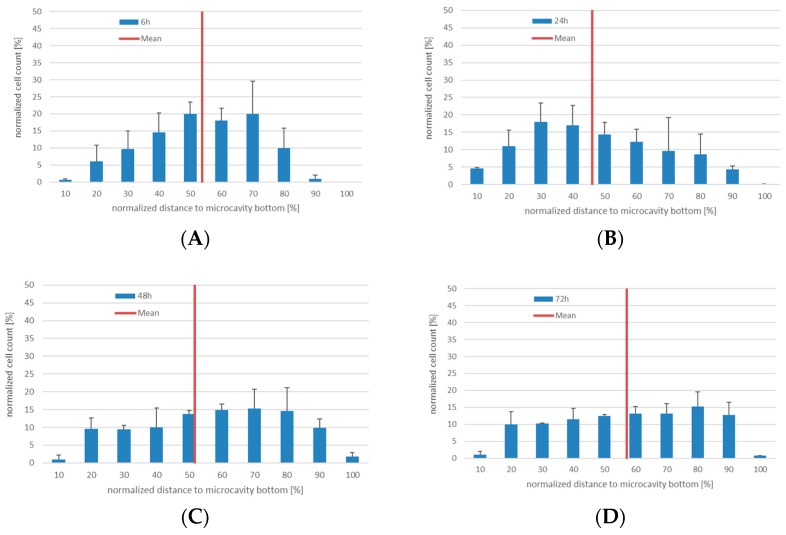
3D co-culture of human bone marrow MSCs with human KG-1a cells in microcavities. Number of CTG^+^-cells and their position relative to the cavity bottom (0%). The mean distance is displayed as a red line, *n* = 3. (**A**) Distribution of KG-1a cells in 3D co-culture after 6 h. (**B**) Distribution of KG-1a cells in 3D co-culture after 24 h. (**C**) Distribution of KG-1a cells in 3D co-culture after 48 h. (**D**) Distribution of KG-1a cells in 3D co-culture after 72 h.

Since the experiments with KG-1a cells in 3D co-culture with Hep G2 cells showed that migration of cells within a microcavity can be detected, we set up a more physiologically-accurate model of the hematopoietic niche. For this, human bone marrow mesenchymal stromal cells in co-culture with KG-1a cells were used. The KG-1a cells were labeled with CTG prior to the inoculation. As before, the cells were cultured for six, 24, 48, and 72 h and the number of proliferating cells as well as their position were determined (Figure 4, the microscope images are shown in Supplementary Figure S3). As in the co-culture of KG-1a together with Hep G2 cells, after 6 hours the labeled cells showed a similar distribution with a median position at 54% ± 7% of the cavity depth. Additionally, the behavior after 24 and 48 h was similar to that observed in the KG-1a/Hep G2 co-culture (median 47% ± 4% and 52% ± 4%). After 72 h a very even distribution within the microcavity could be observed with a median of 57% ± 1%. 

It can be assumed that by changing the co-culture conditions with respect to the niche supporting cells, the KG-1a cells display a different migration behavior. 

**Figure 5:**
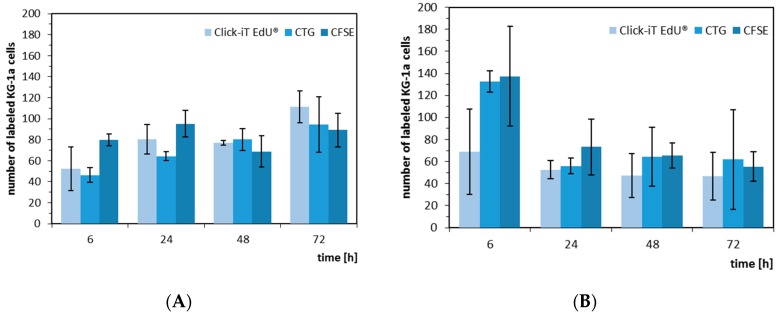
Absolute number of proliferating KG-1a cells in co-culture with Hep G2 (**A**) and hMSC (**B**). The KG-1a cells were labelled with either EdU (light blue), CellTracker^TM^ Green (medium blue), or CFSE (dark blue) and, after labelling, cultivated for 6, 24, 48, and 72 h.

When we analyzed the absolute number of proliferating KG-1a cells in the two co-culture models, we recognized a different behavior of the KG-1a cells. In the Hep G2 co-culture, the cells showed a tendency to an increasing proliferation, whereas in the hMSC co-culture after 24 h a tendency to a more constant proliferation rate was visible, although at six hours there was a discrepancy between the different labeling strategies and labeling of EdU was shown to be most effective when it was performed for two hours since longer labeling resulted in increase of dead cells (Figures S4 and S5). The decrease was more pronounced with CellTracker^TM^ Green and CFSE (Figure 5). This result may indicate a more adult niche behavior even with the promyeloblast CD34^+^ cell type KG-1a. 

### 3.3. Determination of the 4D Cell Count with Antibody Staining against CD34

**Figure 6:**
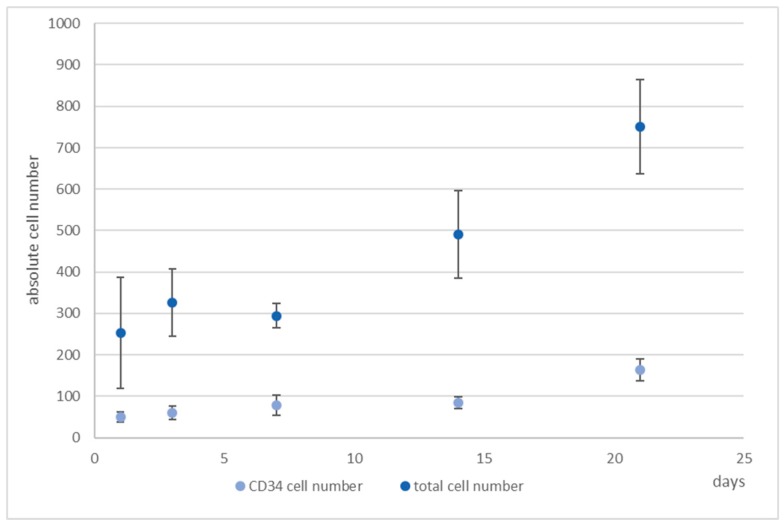
4D cell count of hHSCs and hMSCs in co-culture over the indicated time period after indirect immunofluorescence staining of CD34^+^ cells (**medium blue**) and determination of total cell count (**light blue**) with propidium iodide counterstaining and subsequent software based counting. As can be seen, the total cell number tripled with the ratio of hHSCs:hMSCs kept constant. Used antibodies for CD34^+^ cell detection: CD34 mouse anti-human, ab762, Abcam, and goat-anti-mouse-AlexaFluor488, AC-11001, ThermoFisher Scientific.

Due to the fact that EdU-labeling proved unsuitable for hHSCs (see Supplementary Figures S6 and S7), we detected the hHSCs in microcavities by CTG and/or antibody staining. After antibody staining of the hHSC against CD34, cells were counterstained with propidium iodide and counted. We performed this analysis over a period of up to 21 days. At days 1, 7, 14, and 21 cells were fixed and stained with an antibody against CD34. Over a period of 21 days the total cell numbers of CD34^+^ cells together with the hMSCs tripled, while the ratio kept constant (Figure 6).

**Figure 7:**
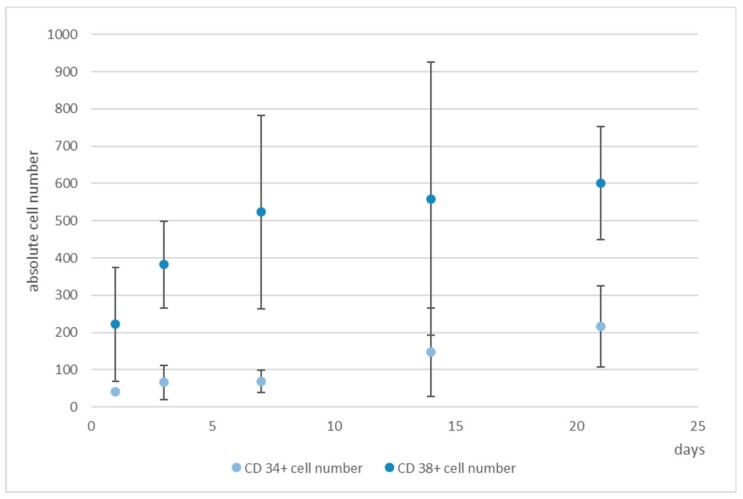
Numbers of CD34^+^ (**light blue**) and CD38^+^ cells (**medium blue**), determined by software-based counting, over a period of 21 days. Used antibodies for CD34 and CD38 cell detection: CD34 mouse anti-human-APC, AC136-APC, ThermoFisher Scientific, and CD38 monoclonal antibody (HIT2), FITC, eBioscience™, ThermoFisher Scientific.

Only 1% of the bone marrow CD34^+^ cells and 0.5% of the cord blood CD34^+^ cells were CD38 negative and were therefore considered as pluripotent hematopoietic stem cells. Increasing CD38 expression indicated the beginning of a differentiation process that can be characterized by the occurrence of myeloblasts, erythroblasts, and lymphoblasts [32]. Moreover, CD34^+^CD38^−^ cells from cord blood were identified as being long term culture initiating cells (LTCIC), meaning that these were able to generate colony foming unit cells (CFU-Cs) in an extended LTCIC-assay after day 60–100, whereas CD34^+^CD38^+^ cells were not able to generate CFU-C beyond day 40 [33]. Since we wanted to get an impression of whether differences in the ratio of CD34^+^/CD38^+^ cells occurred, indicating a loss of stemness, we determined also the number of CD38^+^ cells (Figure 7). As can be seen, whereas the number of CD34^+^ cells increased, the number of CD38^+^ cells hardly changed from day 7 onwards, thereby leading to a change in the ratio of these cells from 1:5 to 1:3 (CD34:CD38). 

### 3.4. Migration Behaviour of hHSCs Inside the Artificial Niche

**Figure 8:**
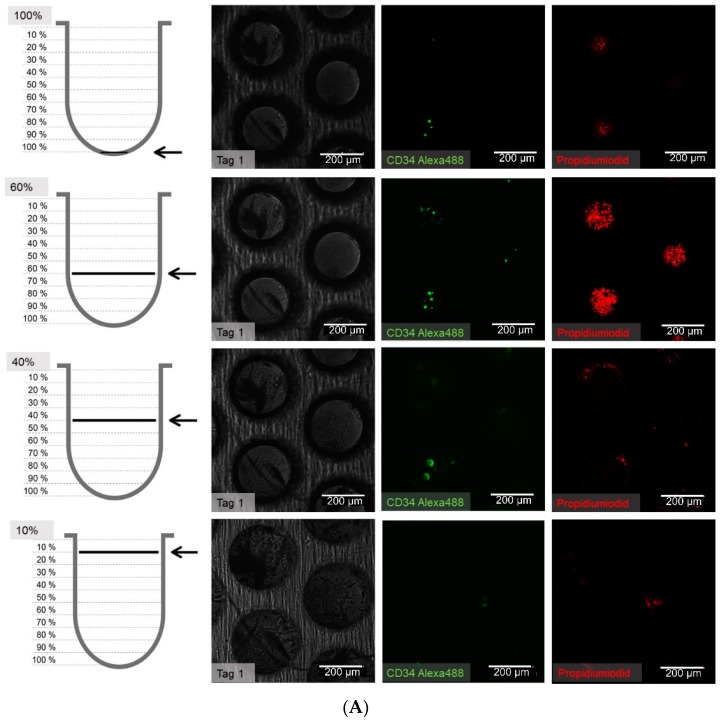

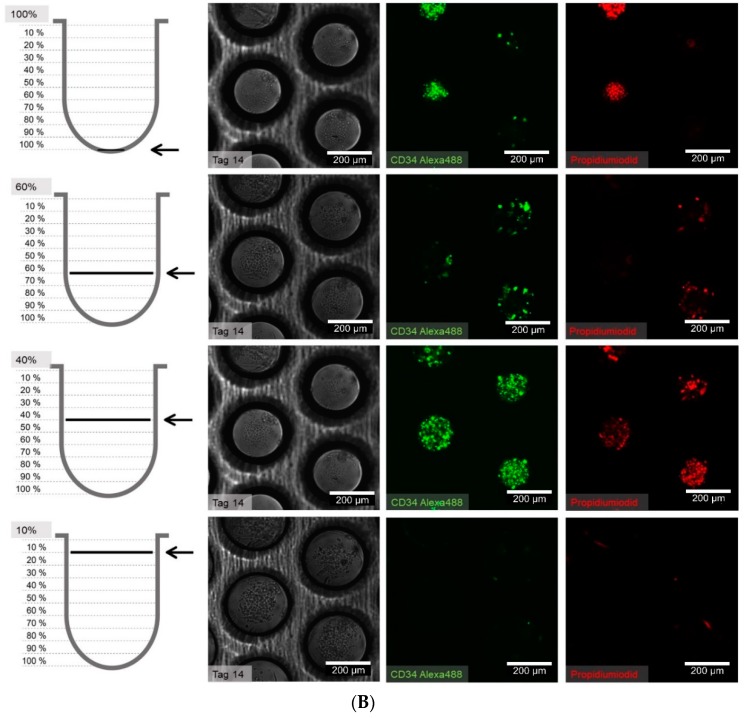

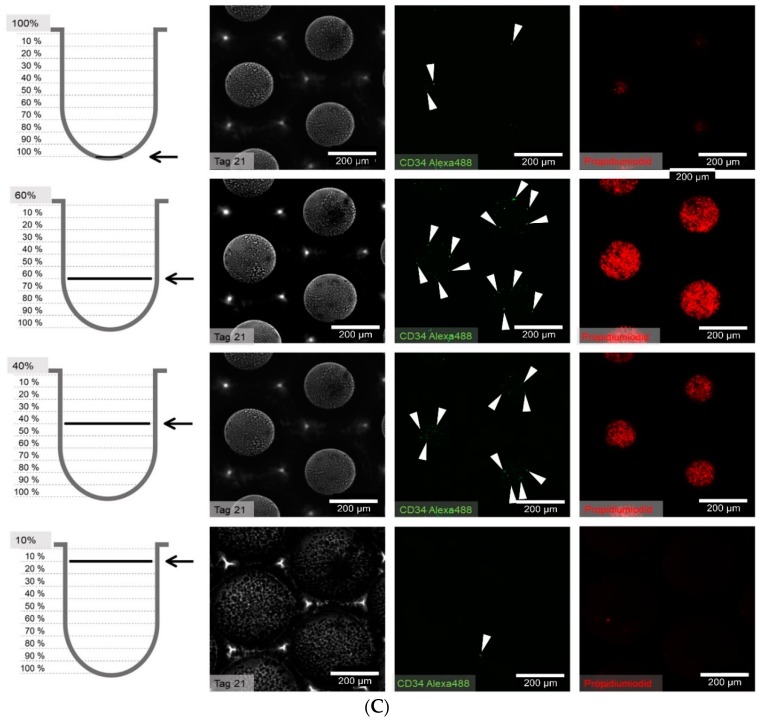
(**A**) Distribution of hHSCs in microcavities after day 1 of co-culture. Scale bar: 200 µm. (**B**) Distribution of hHSCs in microcavities after day 14 of co-culture. (**C**) Distribution of hHSCs in microcavities atfer day 21 of co-culture. White arrowheads point to CD34^+^ cells. Used antibodies for CD34^+^ cell detection: CD34 mouse anti-human, ab762, Abcam, and goat-anti-mouse-AlexaFluor488, AC-11001, ThermoFisher Scientific.

We were then interested in observing the migration behaviour of hHSCs in the static system, since they have a pronounced migratory tendency in the dynamic system [17]. Figure S8 shows the light microscope appearance of the cells and Figure 8A–C shows the fluorescence microscope images of the situation in the microcavities after day 1, 14, and 21. 

**Figure 9:**
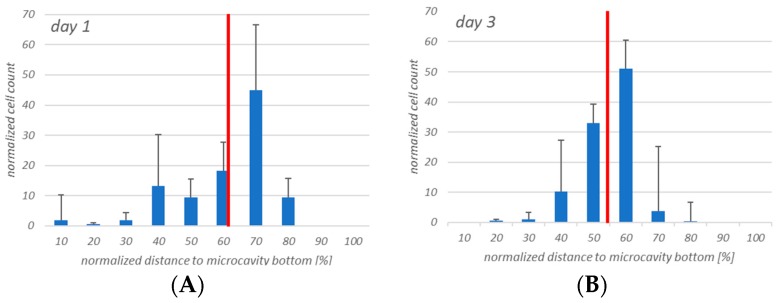

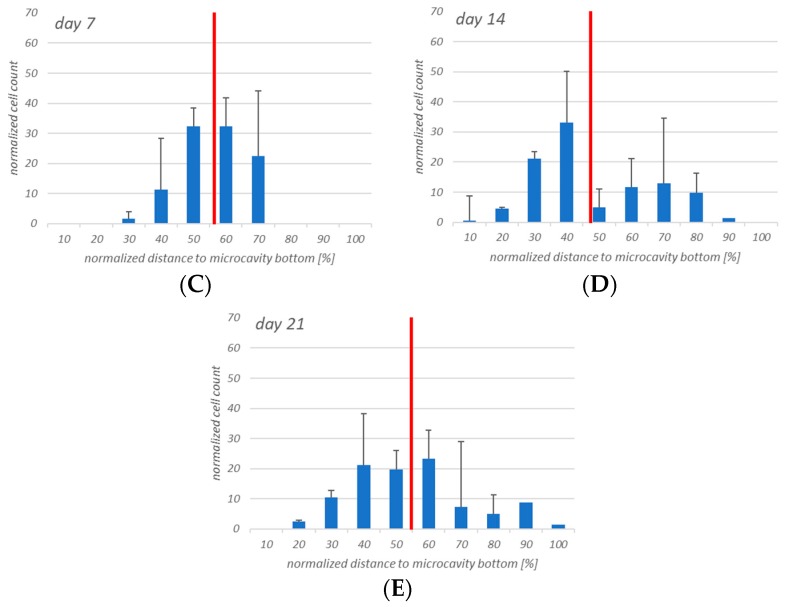
Four-dimensional hHSCs cell count after day 1 (**A**), day 3 (**B**), day 7 (**C**), day 14 (**D**), and day 21 (**E**). Up to day 14 it appears that two distinct populations are formed. However, at day 21 this is less pronounced. Red: Median of the distribution. *n* = 3, * = *p* < 0.05.

The cell counting showed that the hHSCs seemed to segregate into two distinct populations up to day 14: one of these is located towards the middle/top and one resides near the bottom. At day 21 the two populations that formed at day 14 were already mixing again in the middle (Figure 9).

**Figure 10:**
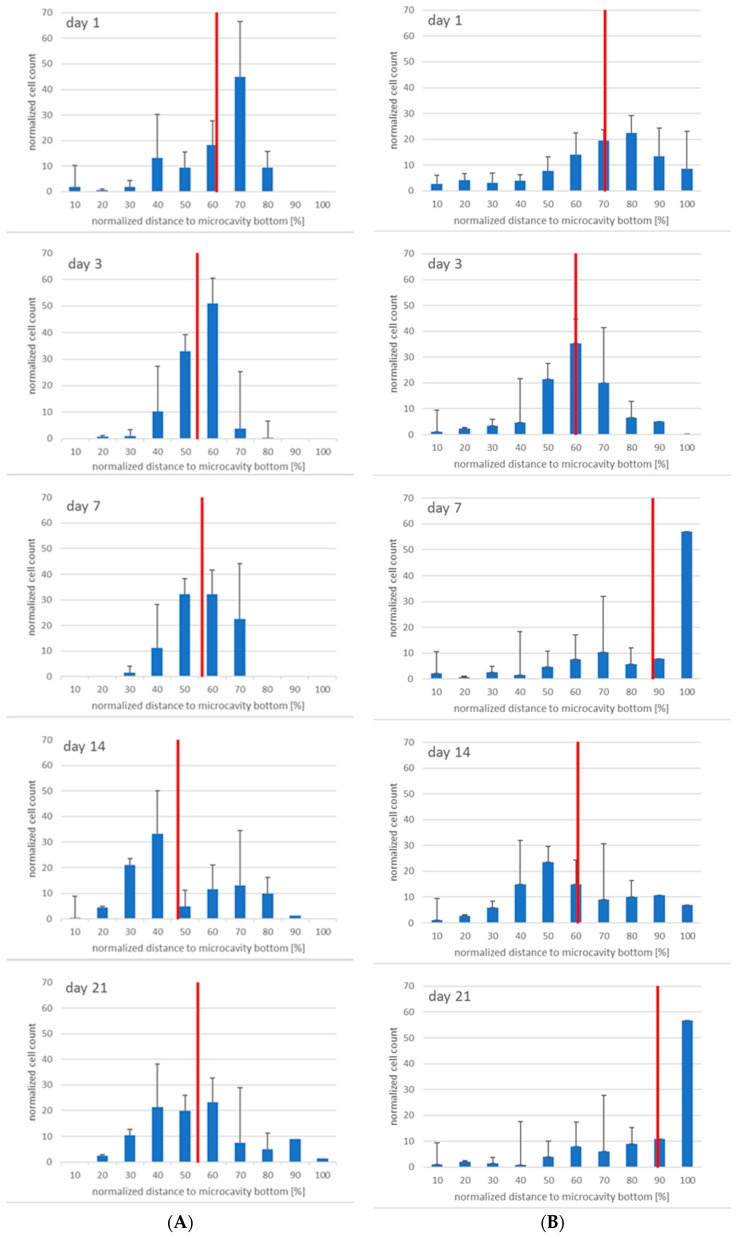
Migratory behaviour of CD34^+^ (**A**) and CD38^+^ (**B**) cells at day 1, 3 7, 14, and 21 (from top to bottom), *n* = 3, * = *p* < 0.05.

Having seen that the number of CD38^+^ cells increased, we were also interested in the three-dimensional distribution of the CD38^+^ cells. Figure 10 shows the results. At days 1 and 3 the populations were more or less congruent but that changed at day 7, when it can be seen that CD34^+^ cells resided more or less around the middle of the cavity while CD38^+^ cells seemed to migrate to the bottom of the cavities. At day 14, there was a tendency of the CD34^+^ cells to segregate into two populations, and this can also be observed for CD38^+^ cells. However, whereas at day 21 the two CD34^+^ populations seem to intermingle, the CD38^+^ cells seem to be found at the bottom of the microcavities, again. 

### 3.5. Flow Cytometric Analysis of the Cellular Niche Composition

Since flow cytometry is capable of quantifiying the number of CD34 and CD38-positive cells we isolated the cells from the microcavities and compared these values of CD34 and CD38-positive cells with those determined by image analysis. Moreover, we compared the 3D with a 2D (petri dish) situation. 

**Figure 11:**
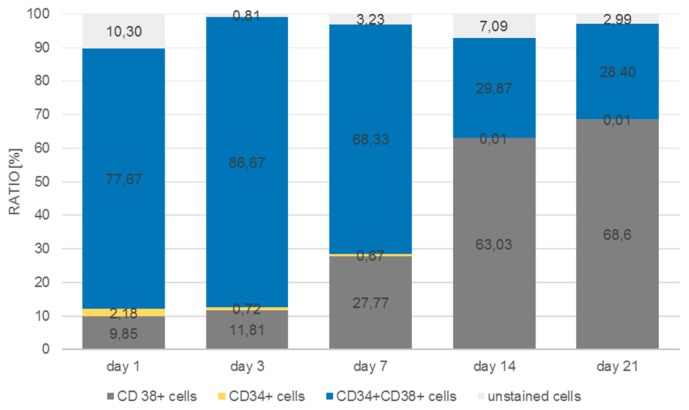
Distribution of CD34^+^, CD38^+^, CD34^+^/CD38^+^, and unstained cells in microcavities over a period of 21 days.

**Figure 12:**
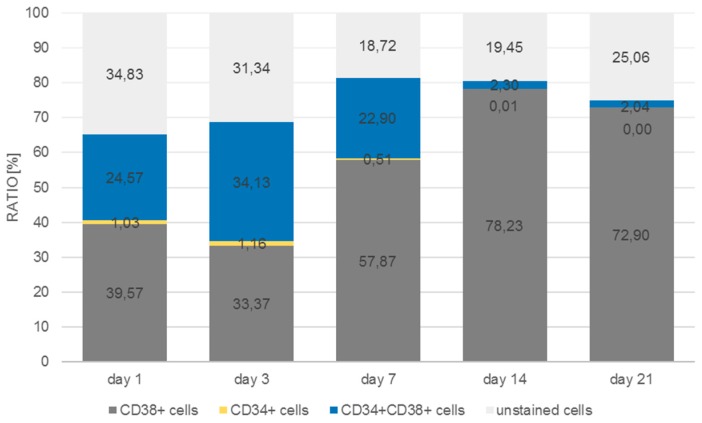
Distribution of CD34^+^, CD38^+^, CD34^+^/CD38^+^, and unstained cells in monolayer over a period of 21 days.

As can be seen in Figure 11, the number of CD38^+^ cells increased from 9.9% to 68.6% of the total cell population measured at day 21, whereas the number of CD34/CD38 double positive cells decreased from 77.7% to 28.4%. Single CD34^+^ cells were detected at day 1 at a percentage of about 2.2% with a tendency to decrease by day 7 to 0.7%, after which they could no longer be detected. In the monolayer (Figure 12) the situation was quite different. The percentage of double-positive cells at day 1 was already much lower (24.6%) and the final number (2%) was dramatically lower than in the 3D situation. Even if we take into account that in the monolayer situation the number of double-positive cells decreased, the decrease in the monolayer amounted up to a factor of 12 whereas the factor in the 3D situation only decreased by 2.7.

However, in contrast to the immunofluorescence staining counts of the 3D model, the number of CD34^+^ cells decreased up to day 7 and only very few CD34^+^ cells could be detected at days 14 and 21, respectively. 

## 4. Discussion

In this paper, we set up a static hematopoietic niche model based on a 96-well microtiter plate equipped with a microcavity array bottom that allowed for the cultivation of 3D aggregates in a defined way and could be used to quantitatively determine the number of dividing cells as well as their position inside single 3D aggregates. For this, we used the CD34 expressing cell line KG-1a as a surrogate of hHSCs in co-culture with either Hep G2 or human bone marrow mesenchymal stromal cells (hMSCs) to establish the methodology. Afterwards, we transferred the method to a co-culture of hHSCs and hMSCs. 

The initial experiments with the KG-1a cells showed that the cells display a migratory behavior within the three-dimensional environment and that this behavior slightly differed between the Hep G2 and the hMSC co-culture. Although the median of the distribution did not differ, there was a tendency of the KG-1a cells to migrate towards the bottom of the microcavity after 72 h of Hep G2-co-culture, whereas in the co-culture with hMSCs there was a very even distribution of the cells throughout the microcavity. Since the environment plays a critical role on the migration of KG-1a cells to migrate [29], it can be assumed that different surface receptor structures on the Hep G2 and hMSC are responsible for this effect. Of these, hepatocyte growth factor receptor (HGFR) and CXC-motive chemokine ligand 12 (CXCL12) are known to promote KG-1a migration after ligand binding [34,35]. Moreover, it has been shown that hMSCs contribute to a stimulation of migration in co-culture with AML cells and that this can be attributed to a CXCL12 and HGF expression [13,34,35,36]. In addition, there was a tendency towards a different proliferation behavior in the two environments with an increased proliferation trend in Hep G2-co-culture and an unchanged proliferation in the hMSC co-culture environment. This might imply that, depending on the cellular composition of the niche, the leukemic stem-like cells react differently. This is supported by data of Francis et al. [37] who showed that KG-1a motility is strongly affected by substrate density and conditioning of the medium: When the plastic surface was coated with fibronectin from 0.5 to 5 µg/cm^2^, the KG-1a cells showed strongest motility at 2.5 µg/cm^2^ onwards and migration was supported best in the mid-exponential growth phase where the medium was conditioned by the cells for several days. Additionally, Beerlage [38] could show that the adhesion of KG-1a cells differs markedly when compared between stromal cells and osteoblasts (CAL72), with the latter being a less attractive adhesion partner for KG-1a. Moreover, KG-1a cells express high levels of CD44 that binds to ligands such as hyaluronic acid, collagens, matrix metalloproteinases, and osteopontin [39]. Since osteopontin is highly expressed in nestin^+^-MSCs, which we have used in this study as well as in our earlier report [17], it can be assumed that KG-1a showed a more pronounced migration behavior in the hMSC co-culture. However, since the proliferation behavior of KG-1a cells in the hMSC environment shows a trend to decrease, even though the migratory behavior was more pronounced, apoptosis may have led to increased cell death in this setup and this will be accounted for in the next experimental series. 

Since the sole availability of the above-mentioned molecules cannot by itself account for the direction of the migration, other factors might also contribute to the migration. One possible factor influencing the migration direction might be concentration gradients of nutrients and/or oxygen that are formed in 3D [40], with the KG-1a traveling along these gradients establishing physiologic oxygen concentration levels. Moreover, it could be observed that in the Hep G2-co-culture model the KG-1a distributed differently within the microcavity. The KG-1a were displaced from the cavity wall by the Hep G2. This might be attributed to the fact that Hep G2 cells show a pronounced tendency to colonize collagen I-treated surfaces, and cells of the same type can organize by adhesion to each other [41,42,43]. Moreover, CXCL12 and HGF are expressed in much lower concentrations than in hMSCs [44,45].

The proliferation of the KG-1a in the two systems also seemed to be different. In the Hep G2 co-culture the data showed a tendency to an increased proliferation rate, whereas in the hMSC co-culture the proliferation was constant with a tendency to decrease, depending on the proliferation assay used. This may also be a consequence of a more stable adhesion of the KG-1a cells, since hMSCs provide a microenvironment that contributes to the maintenance of steady state hematopoiesis [46] and by this may influence proliferation. Another reason for a possibly reduced proliferation of the KG-1a cells is a difference in the local concentration of CO_2_ in the cultures. Although it is known that hypocapnia leads to a decreased proliferation rate of KG-1a cells [47], it seems unlikely that different CO_2_ concentrations could be established in the hMSC co-culture. However, since we did not measure the CO_2_ levels, this remains to be elucidated. 

The antibody staining of the CD34 and CD38-positive cells showed inconsistent results: The distribution of the CD34 and CD38-positive cells was more or less the same at days 1, 3, and 14, whereas at day 7 and 21 CD38^+^ cells seemed to be located at the bottom of the microcavities which would indicate that a clear segregation would have taken place. This does not seem to be very reasonable since cord blood hematopoietic stem cells are double positive for CD34 and CD38 with the exception of a small subpopulation (0.5%) of CD34^+^CD38^−^ cells that are considered to be a rare subpopulation of progenitor cells [33]. Although we could detect this subpopulation with the flow cytometric analysis, it seems unreasonable to assume that the large numbers of CD34^+^ cells in the migration analysis were different from CD34^+^CD38^+^ cells. 

The flow cytometric analysis confirmed that the larger amount of the CD34^+^ cells was also positive for CD38 with only 2% and 1% single positive CD34 cells in the microcavities and the monolayer, respectively. Thus, the software-based detection needs to be improved further. However, what the flow cytometric analysis also showed was that the number of CD34^+^CD38^+^ cells increased over time, indicating that the 3D co-culture environment with mesenchymal stromal cells might be better suited to conserve stem/progenitor features of the cells. With 28% of the cells being double positive at the end of the experiment, the microcavity approach showed a much larger proportion of those cells compared to the control (2D) with only 2%. Even if we take into account that already at day 1 the percentage of double positive cells was much lower than compared to the 3D system (24% vs. 77%), the decrease of the number of these cells was much more pronounced in the 2D system (factor of ~12 compared to a factor ~2.75) indicating that 2D cultures might not reflect appropriate culture conditions for hematopoietic stem/progenitor cells. 

Taken together, we could show that it is, in principle, possible to detect single hematopoietic stem cells in microcavities and track them over 21 days. Since the KG-1a cells displayed a different behaviour in the 3D niche model, it might be assumed that the cellular composition as well three-dimensionality influence the cell behaviour. Although the preliminary data for the hHSC–hMSC co-culture do not show significantly different results, it seems as if the 3D systems displays a better maintenance of the hematopoietic stem cells than the 2D system. However, the microscope analysis of the cells has to be improved further to generate more robust data that can be better compared with, for example, flow cytometry data. 

In principle, it has been shown that the model may be a useful tool in exploring the behavior of different co-culture conditions of various cell types and that it is not limited to two populations. The model may also be extended to experiments including even more cell types, such as perivascular cells or sinusoidal endothelial cells. By this, even more questions can be addressed: Is the number of hHSC niches limited and what might be the minimum niche size of the hematopoietic niche [9]? Is it necessary to expand hHSCs when the number of niches is already limited by nature? Is the success of transplantation a measure of the number of cells engrafting or is it just a reflection of the number of available niches? What is the composition of the niche that supports pluripotent stem cell plasticity in contrast to niches that may support only parts of the hematopoiesis or are there equivalent niches or specialized niches [9]? By varying the cellular composition of the niche and/or the geometry of the microcavities many questions might be addressed by this culture and analysis technique. Last but not least, it has to be taken into consideration that the genealogy of hHSCs leads to pleiotropic effects with regard to cell fate decisions [20] making it also necessary to characterize further the input cell population. 

## Supplementary Materials

The following are available online at https://www.mdpi.com/2306-5354/6/2/50/s1, Figure S1: Confocal image of the co-culture stained with propidium iodide or Hoechst 33342 for nuclei counting with the help of the 3D object counter tool of the Fiji software. Schematic representation of a microcavity, which is divided into ten equal classes for normalization of the cell count per class; Figure S2: 3D-co-culture of KG-1a cells with Hep G2 cells in microcavities after 6 (a), 24 (b), 48 (c), and 72 hours (d). Representative images were taken from 0 % depth (upper left) to 100 % depth (lower right) of the microcavities. Green = KG-1a cells stained with EdU, blue = nuclei stained with Hoechst 33342. Scale bar: 100 µm; Figure S3: 3D-co-culture of KG-1a cells with human bone marrow mesenchymal stromal cells in microcavities after 6 (a), 24 (b), 48 (c), and 72 hours (d). Representative images were taken from 0% depth (upper left) to 100% depth (lower right) of the microcavities. Green = KG-1a cells labelled with EdU, blue = nuclei stained with Hoechst 33342. Scale bar: 100 µm; Figure S4: Percentage ± standard deviation of EdU^+^ KG-1a cells relative to total cell count, *n* = 3; Figure S5: Comparison of the increase in LDH-activity in the culture supernatant to the decrease of the number of EdU^+^ cells over time; Figure S6: hHSCs (red arrows) labeled with EdU for 2 h. Blue = nuclei stained with Hoechst 33342. Scale bar: 100 µm; Figure S7: hHSCs (red arrows) labelled with EdU for 20 h. Blue = nuclei stained with Hoechst 33342. Scale bar: 100 µm; Figure S8: HSC/MSC co-culture appearance after day 1 (a), 3 (b), 7 (c), and 14 (d). Scale bar: 100 µm.

## References

[1] Schofield R. (1978). The relationship between the spleen colony-forming cell and the haemopoietic stem cell. Blood Cells.

[2] Boulais P.E., Frenette P.S. (2015). Making sense of hematopoietic stem cell niches. Blood.

[3] Mikkola H.K., Orkin S.H. (2006). The journey of developing hematopoietic stem cells. Development.

[4] Medvinsky A., Dzierzak E. (1996). Definitive hematopoiesis is autonomously initiated by the AGM region. Cell.

[5] Ivanovs A., Rybtsov S., Welch L., Anderson R.A., Turner M.L., Medvinsky A. (2011). Highly potent human hematopoietic stem cells first emerge in the intraembryonic aorta-gonad-mesonephros region. J. Exp. Med..

[6] Morrison S.J., Spradling A.C. (2008). Stem cells and niches: Mechanisms that promote stem cell maintenance throughout life. Cell.

[7] Kanji S., Pompili V.J., Das H. (2011). Plasticity and maintenance of hematopoietic stem cells during development. Recent Pat. Biotechnol..

[8] Kiel M.J., Morrison S.J. (2008). Uncertainty in the niches that maintain haematopoietic stem cells. Nat. Rev. Immunol..

[9] Ugarte F., Forsberg E. (2013). Haematopoietic stem cell niches: New insights inspire new questions. Embo J..

[10] Passegue E., Wagers A.J., Giuriato S., Anderson W.C., Weissman I.L. (2005). Global analysis of proliferation and cell cycle gene expression in the regulation of hematopoietic stem and progenitor cell fates. J. Exp. Med..

[11] Adamo L., Naveiras O., Wenzel P.L., McKinney-Freeman S., Mack P.J., Gracia-Sancho J., Suchy-Dicey A., Yoshimoto M., Lensch M.W., Yoder M.C. (2009). Biomechanical forces promote embryonic haematopoiesis. Nature.

[12] Wang L.D., Wagers A.J. (2011). Dynamic niches in the origination and differentiation of haematopoietic stem cells. Nat. Rev. Mol. Cell Biol..

[13] Jing D., Fonseca A.V., Alakel N., Fierro F.A., Muller K., Bornhauser M., Ehninger G., Corbeil D., Ordemann R. (2010). Hematopoietic stem cells in co-culture with mesenchymal stromal cells--modeling the niche compartments in vitro. Haematologica.

[14] Tan J., Liu T., Hou L., Meng W., Wang Y., Zhi W., Deng L. (2010). Maintenance and expansion of hematopoietic stem/progenitor cells in biomimetic osteoblast niche. Cytotechnology.

[15] Kurth I., Franke K., Pompe T., Bornhauser M., Werner C. (2009). Hematopoietic stem and progenitor cells in adhesive microcavities. Integr. Biol. (Camb.).

[16] Cook M.M., Futrega K., Osiecki M., Kabiri M., Kul B., Rice A., Atkinson K., Brooke G., Doran M. (2012). Micromarrows--three-dimensional coculture of hematopoietic stem cells and mesenchymal stromal cells. Tissue Eng. Part C Methods.

[17] Wuchter P., Saffrich R., Giselbrecht S., Nies C., Lorig H., Kolb S., Ho A.D., Gottwald E. (2016). Microcavity arrays as an in vitro model system of the bone marrow niche for hematopoietic stem cells. Cell Tissue Res..

[18] Muller E., Grinenko T., Pompe T., Waskow C., Werner C. (2015). Space constraints govern fate of hematopoietic stem and progenitor cells in vitro. Biomaterials.

[19] Lutolf M.P., Doyonnas R., Havenstrite K., Koleckar K., Blau H.M. (2009). Perturbation of single hematopoietic stem cell fates in artificial niches. Integr. Biol. (Camb.).

[20] Kokkaliaris K.D., Drew E., Endele M., Loeffler D., Hoppe P.S., Hilsenbeck O., Schauberger B., Hinzen C., Skylaki S., Theodorou M. (2016). Identification of factors promoting ex vivo maintenance of mouse hematopoietic stem cells by long-term single-cell quantification. Blood.

[21] Wenzel C., Otto S., Prechtl S., Parczyk K., Steigemann P. (2015). A novel 3D high-content assay identifies compounds that prevent fibroblast invasion into tissue surrogates. Exp. Cell Res..

[22] Giselbrecht S., Gietzelt T., Gottwald E., Guber A., Trautmann C., Truckenmüller R., Weibezahn K.-F. (2004). Microthermoforming as a novel technique for manufacturing scaffolds in tissue engineering. IEE Proc. Nanobiotechnol..

[23] Giselbrecht S., Gietzelt T., Gottwald E., Trautmann C., Truckenmüller R., Weibezahn K.-F., Welle A. (2006). 3D tissue culture substrates produced by microthermoforming of pre-processed polymer films. Biomed. Microdev..

[24] Truckenmüller R., Giselbrecht S., van Bitterswijk C., Dambrowsky N., Gottwald E., Mappes T., Rolletschek A., Saile V., Trautmann C., Weibezahn K.-F. (2008). Flexible fluidic microchips based on thermoformed and locally modified thin polymer films. Lab Chip.

[25] Truckenmuller R., Giselbrecht S., Rivron N., Gottwald E., Saile V., Van den Berg A., Wessling M., Van Blitterswijk C. (2010). Thermoforming of film-based biomedical microdevices. Adv. Mater..

[26] Giselbrecht S., Gottwald E., Truckenmüller R., Trautmann C., Welle A., Guber A., Saile V., Gietzelt T., Weibezahn K.-F. (2008). Microfabrication of chip-sized scaffolds for the three-dimensional cell cultivation. JoVE.

[27] Truckenmüller R., Rummler Z., Schaller T., Schomburg W.K. (2002). Low-cost thermoforming of micro fluidic analysis chips. J. Micromech. Microeng..

[28] Truckenmüller R., Giselbrecht S. (2004). Microthermoforming of flexible, not buried hollow microstructures for chip-based life sciences applications. IEE Proc. Nanobiotechnol..

[29] Francis K., Palsson B., Donahue J., Fong S., Carrier E. (2002). Murine Sca-1(+)/Lin(−) cells and human KG1a cells exhibit multiple pseudopod morphologies during migration. Exp. Hematol..

[30] Schindelin J., Arganda-Carreras I., Frise E., Kaynig V., Longair M., Pietzsch T., Preibisch S., Rueden C., Saalfeld S., Schmid B. (2012). Fiji: An open-source platform for biological-image analysis. Nat. Methods.

[31] Bolte S., Cordelieres F.P. (2006). A guided tour into subcellular colocalization analysis in light microscopy. J. Microsc..

[32] Terstappen L.W., Huang S., Safford M., Lansdorp P.M., Loken M.R. (1991). Sequential generations of hematopoietic colonies derived from single nonlineage-committed CD34+CD38- progenitor cells. Blood.

[33] Hao Q.L., Shah A.J., Thiemann F.T., Smogorzewska E.M., Crooks G.M. (1995). A functional comparison of CD34 + CD38- cells in cord blood and bone marrow. Blood.

[34] Donepudi M.S., Kondapalli K., Amos S.J., Venkanteshan P. (2014). Breast cancer statistics and markers. J. Cancer Res. Ther..

[35] Sison E.A., Brown P. (2011). The bone marrow microenvironment and leukemia: Biology and therapeutic targeting. Expert Rev. Hematol..

[36] Neuss M., Roser M., Kelle S., Girke F., Winbeck G., Stawowy P., Fleck E. (2004). Calculation of individual cardiovascular risk in a primary prevention setting shows a high number of patients with a high risk score. Z. Kardiol..

[37] Francis K., Lee G.M., Palsson B.O. (2002). Characterization of the KG1a Cell Line for Use in a Cell Migration Based Screening Assay. Biotechnol. Bioprocess. Eng..

[38] Beerlage A. (2016). Interaktion von Tumorzellen in der hämatopoetischen Stammzellnische.

[39] Senbanjo L.T., Chellaiah M.A. (2017). CD44: A Multifunctional Cell Surface Adhesion Receptor Is a Regulator of Progression and Metastasis of Cancer Cells. Front. Cell Dev. Biol..

[40] Kenney R.M., Lloyd C.C., Whitman N.A., Lockett M.R. (2017). 3D cellular invasion platforms: How do paper-based cultures stack up?. Chem. Commun..

[41] Wilkening S., Stahl F., Bader A. (2003). Comparison of primary human hepatocytes and hepatoma cell line Hepg2 with regard to their biotransformation properties. Drug Metab. Dispos..

[42] Bouma M.E., Rogier E., Verthier N., Labarre C., Feldmann G. (1989). Further cellular investigation of the human hepatoblastoma-derived cell line HepG2: Morphology and immunocytochemical studies of hepatic-secreted proteins. In Vitro Cell. Dev. Biol..

[43] Townes P.L., Holtfreter J. (1955). Directed movements and selective adhesion of embryonic amphibian cells. J. Exp. Zool..

[44] Michalopoulos G.K., Zarnegar R. (1992). Hepatocyte growth factor. Hepatology.

[45] Nagamine T., Hayakawa K., Kusakabe T., Takada H., Nakazato K., Hisanaga E., Iha M. (2009). Inhibitory effect of fucoidan on Huh7 hepatoma cells through downregulation of CXCL12. Nutr. Cancer.

[46] Majumdar M.K., Keane-Moore M., Buyaner D., Hardy W.B., Moorman M.A., McIntosh K.R., Mosca J.D. (2003). Characterization and functionality of cell surface molecules on human mesenchymal stem cells. J. Biomed. Sci..

[47] Hamad M., Irhimeh M.R., Abbas A. (2016). Hypercapnia slows down proliferation and apoptosis of human bone marrow promyeloblasts. Bioprocess Biosyst. Eng..

