# Supplementary material for: A Microcavity Array-Based 4D Cell Culture Platform
Source: Bioengineering (Basel). 2019 May 31;6(2):50. doi: 10.3390/bioengineering6020050 (PMC6631836; doi:10.3390/bioengineering6020050)
Supplement: Supplementary file 1 [file bioengineering-06-00050-s001.pdf]

Supplementary Materials to "A microcavity array based 4D cell culture platform"

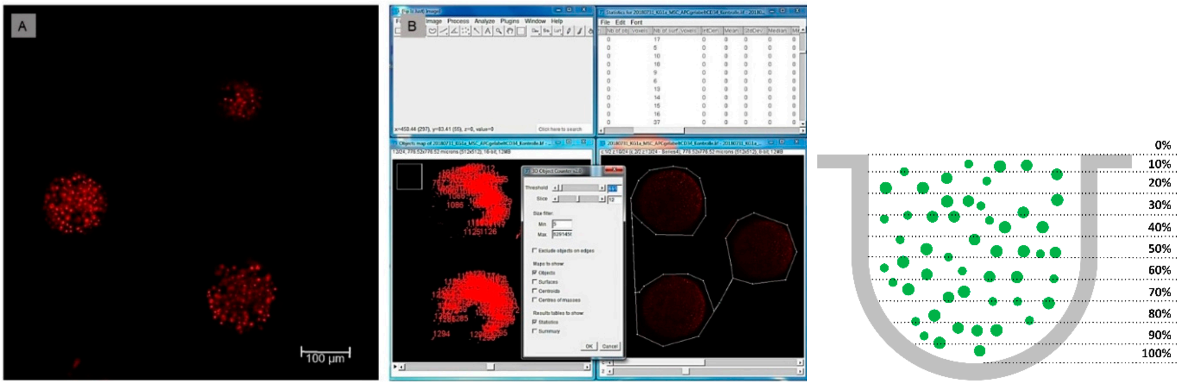

Figure S1. Confocal image of the co-culture stained with propidium iodide or Hoechst 33342 for nuclei counting with the help of the 3D object counter tool of the Fiji software. Schematic representation of a microcavity, which is divided into ten equal classes for normalization of the cell count per class.

(a)

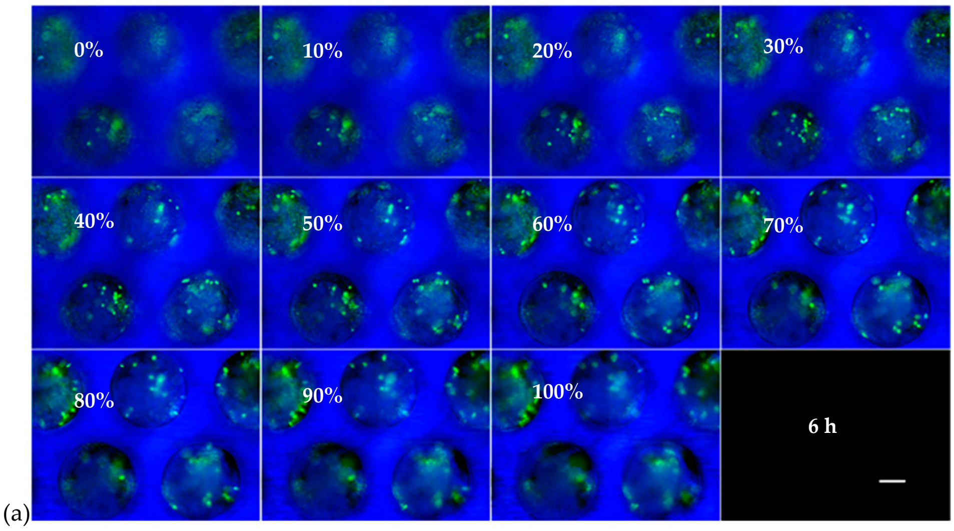

(b)

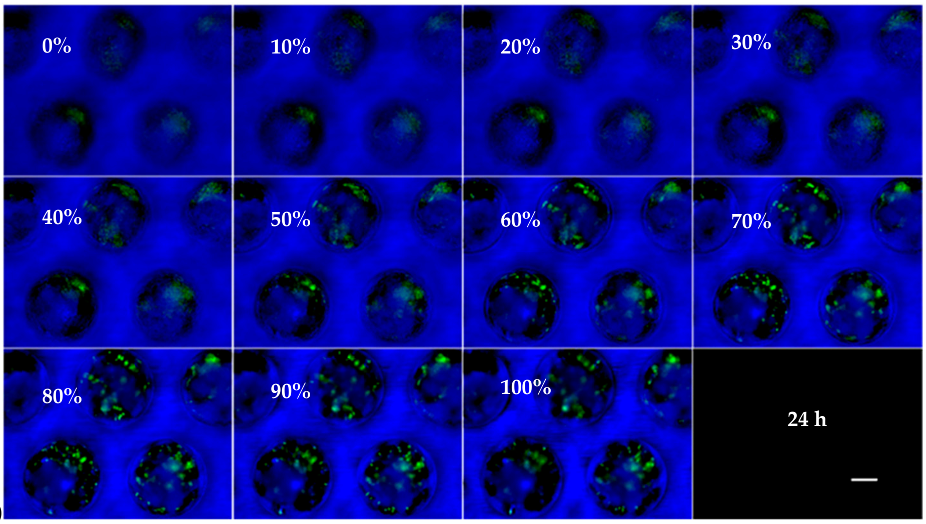

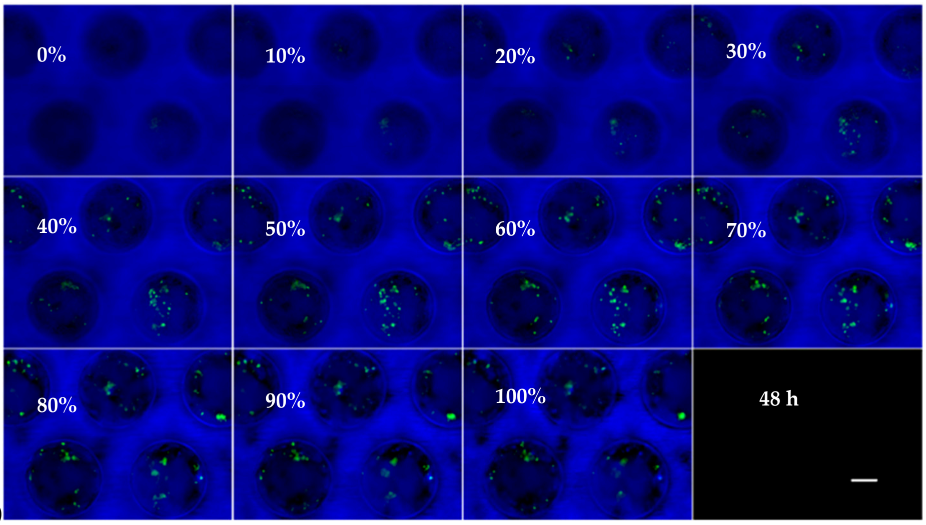

(c)

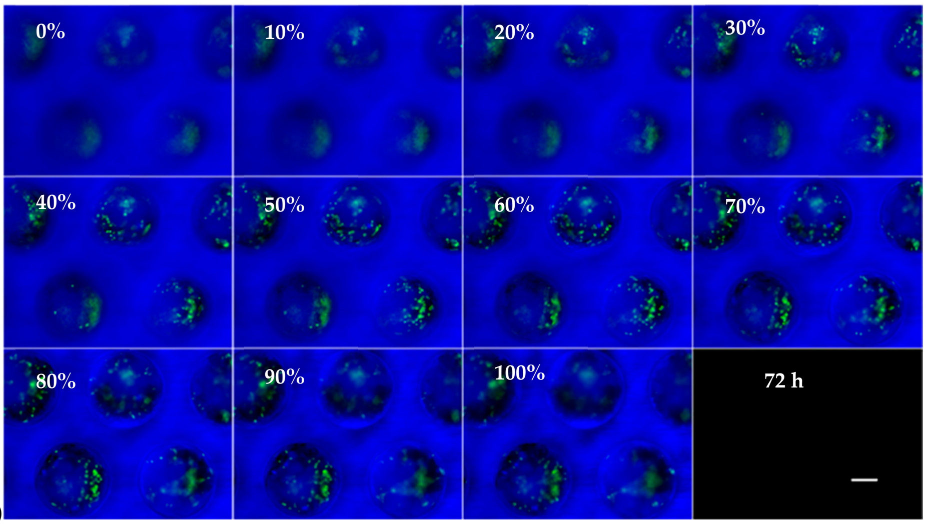

(d)

Figure S2. 3D-co-culture of KG-1a cells with Hep G2 cells in microcavities after 6 (a), 24 (b), 48 (c), and 72 hours (d). Representative images were taken from 0 % depth (upper left) to 100 % depth (lower right) of the microcavities. Green = KG-1a cells stained with EdU, blue = nuclei stained with Hoechst 33342. Scale bar: 100 µm.

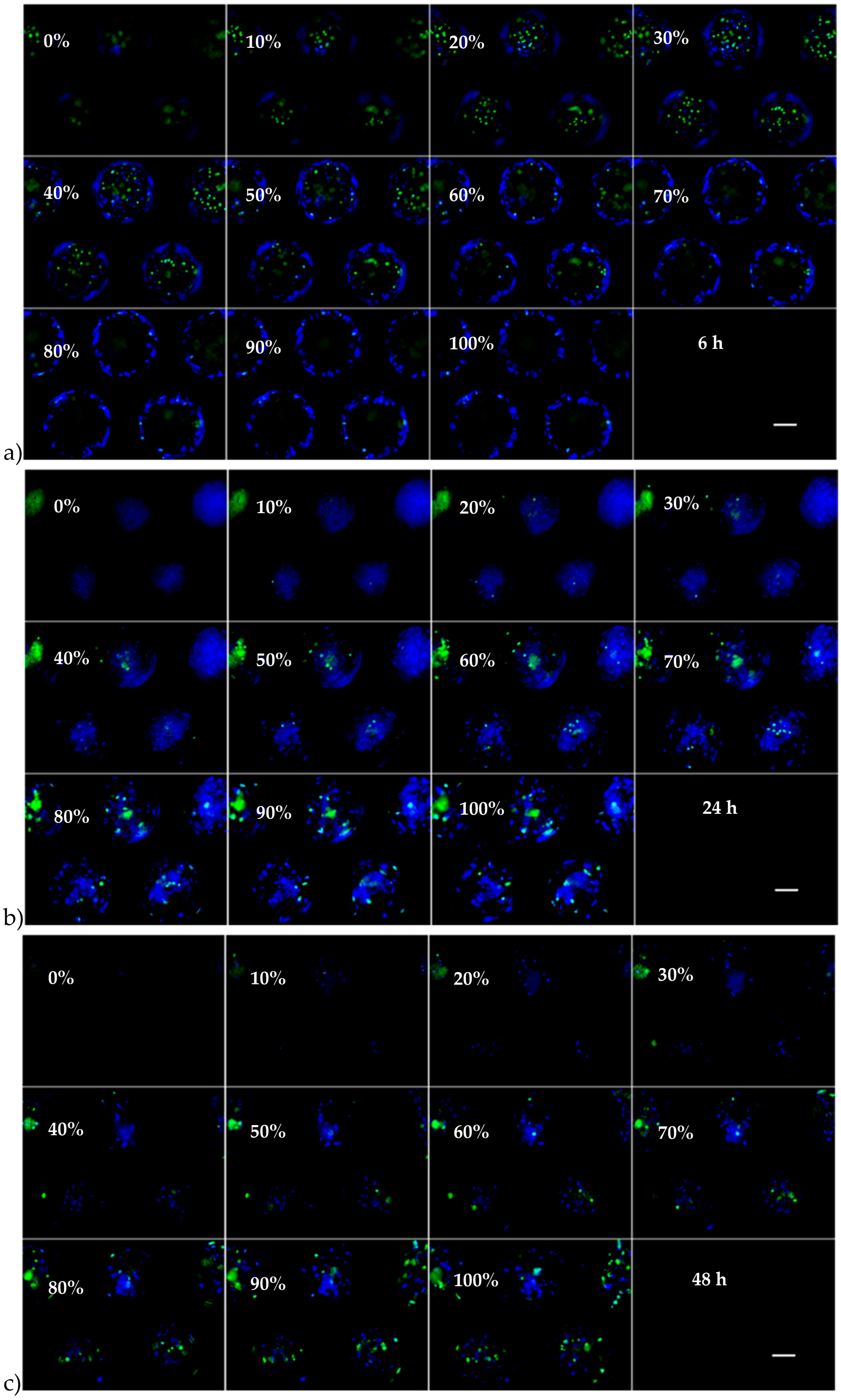

0%
10%
20%
30%
40%
50%
60%
70%
80%
90%
100%
6 h
a)
0%
10%
20%
30%
40%
50%
60%
70%
80%
90%
100%
24 h
b)
0%
10%
20%
30%
40%
50%
60%
70%
80%
90%
100%
48 h
c)

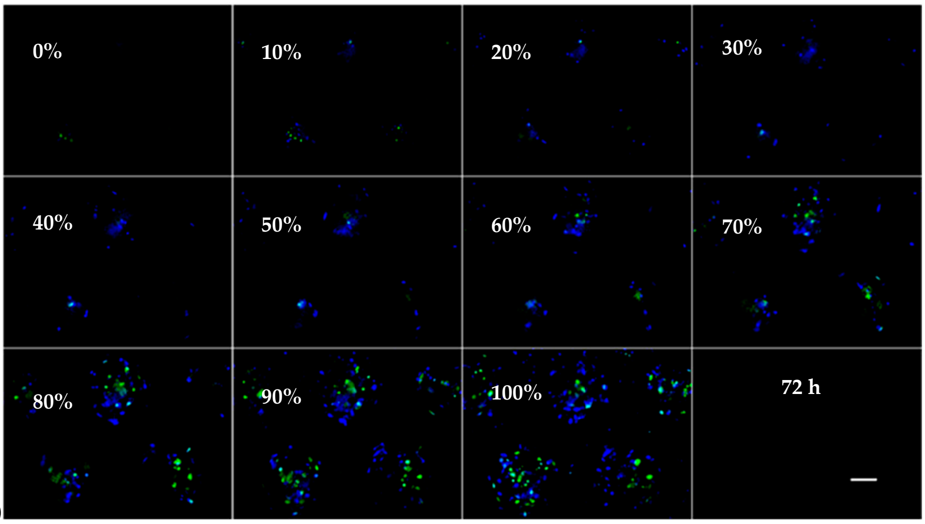

d)

Figure S3. 3D-co-culture of KG-1a cells with human bone marrow mesenchymal stromal cells in microcavities after 6 (a), 24 (b), 48 (c), and 72 hours (d). Representative images were taken from 0% depth (upper left) to 100% depth (lower right) of the microcavities. Green = KG-1a cells labelled with EdU, blue = nuclei stained with Hoechst 33342. Scale bar: 100 µm.

Labeling of KG-1a with EdU in suspension culture

Since the labeling efficiency seemed to drop after longer cultivation times we labelled KG-1a cells after various time points of suspension culture (2, 24, 48 and 72 h). As shown in Fig. 12, the labeling efficiency (EdU+ cell fraction of total cell count) of KG-1a cells drops from an initial value of 43 % ± 3 % to 37 % ± 3 % after 24 h, 25 % ± 1 % after 48 h and 14 % ± 1 % after 72 h of culture. Since it is known that EdU may be toxic to cells, we performed an LDH-assay to detect possible cell death associated with the labeling procedure.

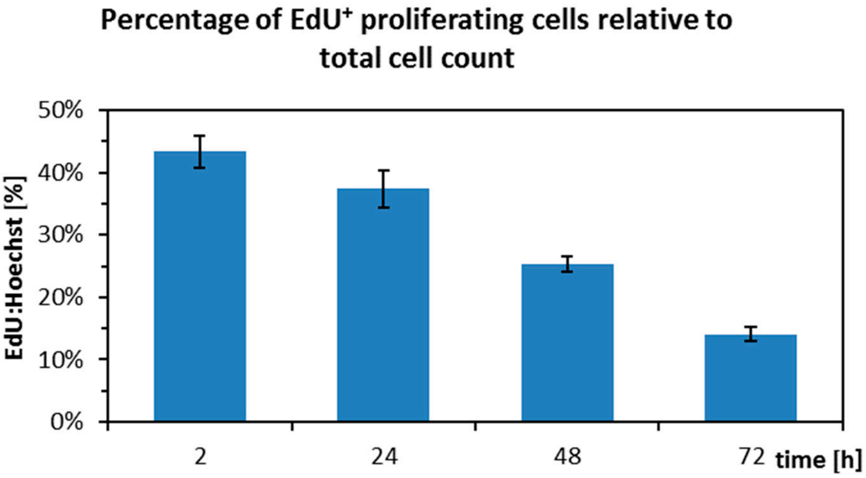

Figure S4. Percentage ± standard deviation of EdU^+ KG-1a cells relative to total cell count, $n = 3$.

Since KG-1a cells are not reported to display a density/contact inhibition phenomenon, we were asking for the reason of the detected decreasing labeling efficiency. Therefore, we performed a LDH-assay to detect a possible toxicity of the EdU and compared the decrease in labeling efficiency with the increase of LDH in the culture supernatant (Fig. S5).

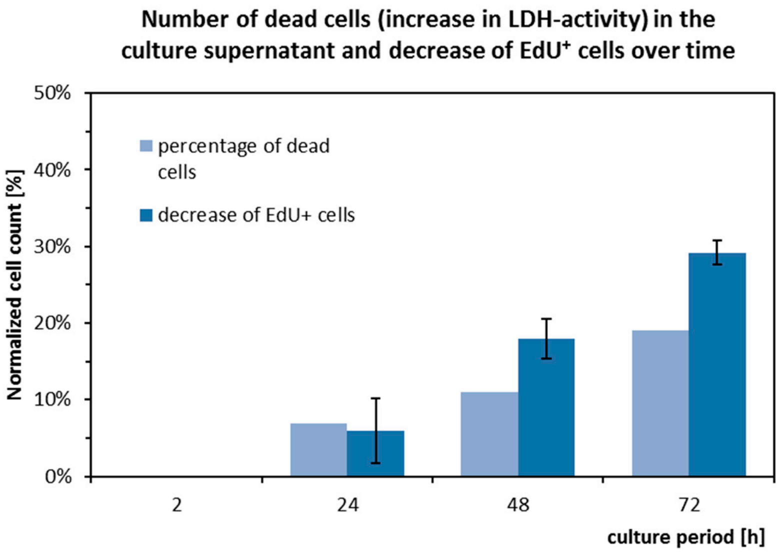

Figure S5. Comparison of the increase in LDH-activity in the culture supernatant to the decrease of the number of EdU^+ cells over time.

When the cells were incubated for 2 h with EdU and cultivated for 2, 24 , 48, and 72 hours respectively, we detected a decrease in labeling efficiency that was paralleled by an increase in LDH-activity in the culture supernatant. Thus, the EdU may display a cytotoxic effect in our system.

EdU-labeling experiments with hHSCs

The established technology of labelling cells with EdU prior to introduction into the microcavity and concomittant microscope detection was then transferred to an hHSC-hMSC co-culture. The appearance of the co-culture after days 1, 3, 7, and 14 is depicted in supplementary fig. S6. As before, EdU-labelling was performed for 2 hours and afterwards mixed in suspension with hMSCs. The microscope detection revealed that the HSCs were only minimally labeled (Fig. S6), indicating their slow cell cycle.

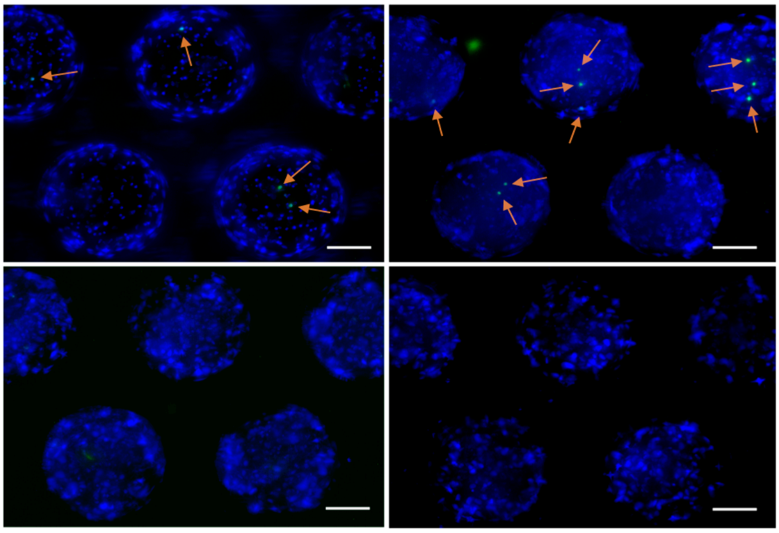

Figure S6. hHSCs (red arrows) labeled with EdU for 2 h. Blue = nuclei stained with Hoechst 33342. Scale bar: 100 µm.

We therefore extended the labelling duration to 20 hours and repeated the experiment. As can be seen in Fig. S7, despite the ten times longer labelling period only a very limited fraction of the

hHSCs was labelled, which meant that a longer observation and quantitative analysis of the migration and proliferation behaviour was not possible. For the slow cycling population of hHSCs it is clear that a different labelling strategy has to be applied.

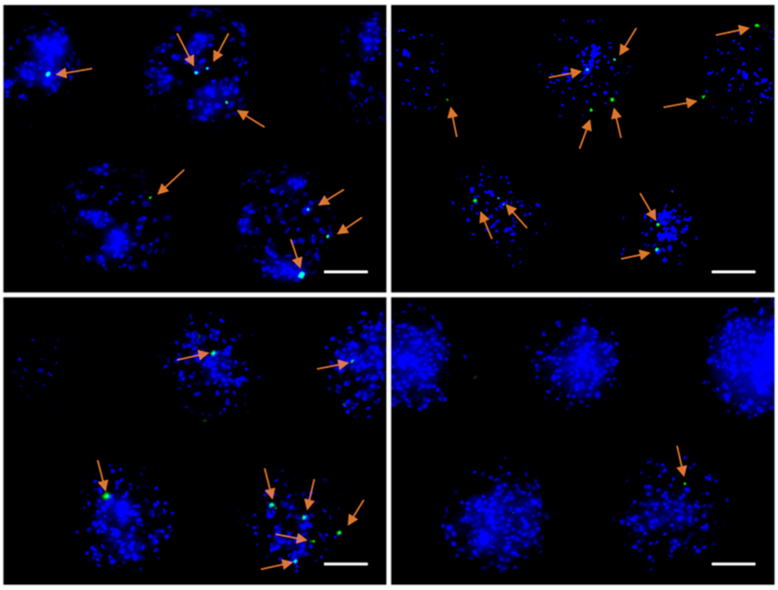

Figure S7. hHSCs (red arrows) labelled with EdU for 20 h. Blue = nuclei stained with Hoechst 33342. Scale bar: 100 µm.

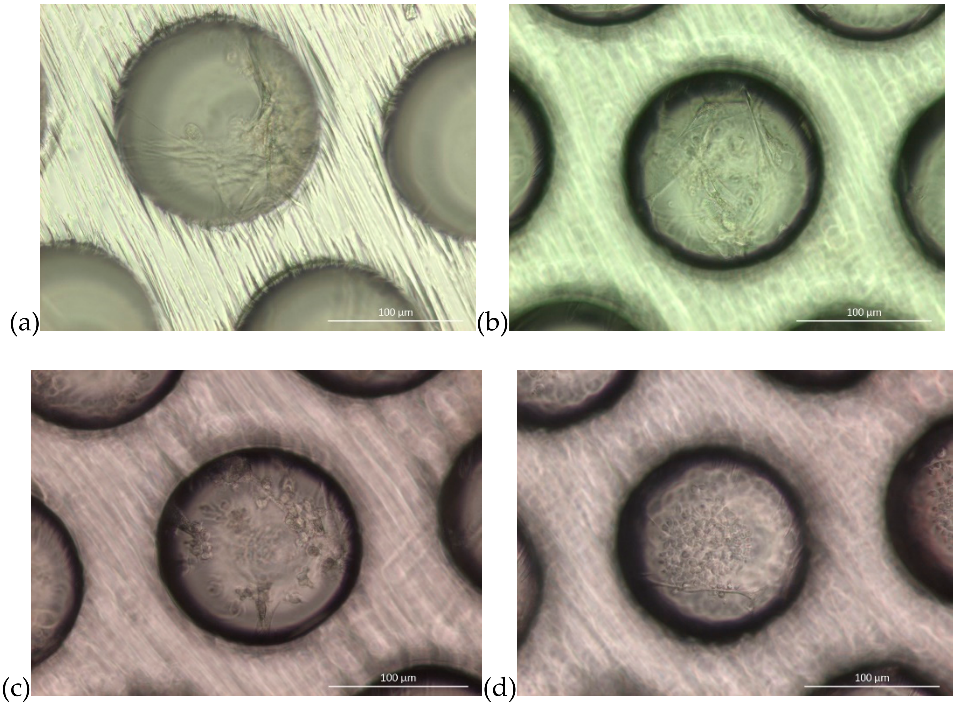

Figure S8. HSC/MSC co-culture appearance after day 1 (a), 3 (b), 7 (c), and 14 (d). Scale bar: 100 µm.
